# Multi-Omics Analysis of Chronic Heat Stress-Induced Biological Effects, Liver Injury, and Heat Tolerance Mechanisms via Oxidative and Anti-Inflammatory Pathways in Early-Pregnancy Sows

**DOI:** 10.3390/antiox14060623

**Published:** 2025-05-23

**Authors:** Jie Chai, Zhenhao Wen, Li Chen, Qiang Pu, Taorun Luo, Xiaoqian Wu, Zihan Ma, Zonggang Luo, Jia Luo, Jingyong Wang

**Affiliations:** 1 Chongqing Academy of Animal Sciences, Chongqing 402460, China; chaijie@cqaa.cn (J.C.); chenli@cqaa.cn (L.C.); 2 College of Animal Science and Technology, Southwest University, Chongqing 400715, China; w15136316696@email.swu.edu.cn (Z.W.); pq012024328000466@email.swu.edu.cn (Q.P.); cristianoltr@email.swu.edu.cn (T.L.); zh911005@email.swu.edu.cn (Z.M.); luozonggang@swu.edu.cn (Z.L.); 3 College of Food Science, Southwest University, Chongqing 400715, China; 517@email.swu.edu.cn

**Keywords:** heat stress, liver, pregnant, oxidative stress, sow

## Abstract

The prenatal environment critically influences sow and offspring health, with the liver being highly susceptible to heat stress (HS) and vital for antioxidant defense. However, mechanisms underlying HS impacts on early pregnancy and hepatic adaptation remain unclear. This study applied multi-omics to analyze chronic HS responses in early-pregnancy sows. Results demonstrated that HS reduced blood oxygen (PO_2_) and basophils while elevating red blood cell parameters (RBC, HGB, and HCT). Endocrine disruptions included upregulated adrenal hormones (ACTH and cortisol) and suppressed thyroid (T3 and TSH) and reproductive hormones (LH1 and FSH). Liver dysfunction was evident through elevated biomarkers (AST, ALT, and TBIL) and pro-inflammatory IL-6, coupled with reduced anti-inflammatory IL-10. HS induced oxidative stress, marked by increased total antioxidant capacity (T-AOC) but decreased SOD and MDA levels. Liver tissue exhibited apoptosis (Bax/CD8 upregulated and Bcl-2 downregulated) and upregulated heat shock proteins (HSP70/90). Multi-omics analysis demonstrated that under heat stress conditions, the pyrimidine metabolism, oxidative phosphorylation, and tryptophan metabolism pathways were significantly upregulated in the liver. This upregulation may be mediated by key metabolites, including AMP, NAD, and UMP. These metabolites likely contribute to the body’s adaptation to heat stress. Chronic HS impaired liver function and anti-inflammatory responses but triggered compensatory antioxidant and metabolic reprogramming. These findings underscore the liver’s dual characteristics of vulnerability and resilience under high-temperature stress, offering valuable mechanistic insights that can inform strategies to enhance heat tolerance in pregnant sows.

## 1. Introduction

Global temperatures are escalating at an unprecedented rate, with an average surface temperature increase of 0.2 °C ± 0.1 °C per decade [1]. The rising frequency and intensity of heat events pose significant threats to human survival and livestock production [2], particularly endangering the health of pregnant women and animals [3,4]. Compared to other species, pigs exhibit underdeveloped sweat gland systems that lack functional sweat glands, rendering their thermoregulatory mechanisms highly susceptible to high-temperature environments [5,6]. Early gestation represents a pivotal phase for porcine embryo establishment and development, characterized by acute sensitivity to environmental stressors [7]. During this developmental window, fetal vulnerability to thermal damage escalates significantly, correlating with a heightened incidence of congenital malformations [8]. The liver, a critical metabolic organ, demonstrates heightened sensitivity to heat stress (HS), which triggers severe hepatic injury [9,10]. Previous studies have found that periodic chronic heat stress triggers oxidative stress and inflammation in the liver, leading to liver cell damage [11]. In pregnant sows, HS significantly compromises reproductive performance by inducing hepatic oxidative stress, which in turn leads to metabolic dysregulation, reproductive dysfunction, elevated fetal mortality, spontaneous abortion, and immunosuppression [12,13]. Furthermore, HS disrupts the intrauterine environment, diminishing embryo survival rates and reducing birth weights—key economic indicators of production efficiency—ultimately driving substantial economic losses in global livestock industries [14,15].

Current genetic selection has reduced pigs’ heat stress resistance. Modern commercial pig breeds, selected for high growth and reproduction, produce more metabolic heat but have weaker adaptive capacity, worsening the impact of heat stress, especially during pregnancy and lactation [16,17]. Pregnant sows respond differently to ambient temperatures depending on their gestational stage. Non-pregnant sows experience heat stress above 25.5 °C, mid-gestation sows at 25.1 °C, and late-gestation sows at 24.0 °C. Severe heat stress occurs at temperatures over 32.9 °C in the second trimester and 30.8 °C in the third trimester [18]. Relevant studies propose adaptive strategies to reduce climate change impacts on livestock production and economic losses [19,20]. However, these measures are costly, with significant economic and environmental burdens. Recent studies have demonstrated that the heritability of productive traits is modulated by environmental factors, with a notable genetic correlation observed between the temperature–humidity index (THI) and key reproductive traits such as the total number born (TNB), number born alive (NBA), and stillbirth rate (STBR) [21]. Incorporating the THI into breeding value assessments can help identify pigs with greater resistance to heat stress. Consequently, a comprehensive understanding of the biological effects and mechanisms of heat stress on sows will enhance their environmental adaptability and production efficiency, providing a robust scientific basis for improved production management.

Currently, research into the molecular mechanisms underlying heat stress adaptation in early-pregnancy sows remains relatively limited. However, to date, there remains a notable gap in multi-omics research that integrates transcriptomics, proteomics, and metabolomics to elucidate the physiological responses of early-pregnancy sows under heat stress. Particularly, the specific heat stress response of the liver in pregnant sows has yet to be fully understood. Multi-omics approaches have been extensively utilized to integrate data at the gene, protein, and metabolite levels, thereby providing deeper insights into how organisms adapt to environmental stress. Rongchang pigs, a distinguished local breed in China, have evolved unique tolerance to heat and humidity over three centuries of natural selection and adaptive evolution. This enables them to maintain high reproductive performance in a subtropical monsoon climate characterized by an average annual temperature of 18.4 °C and sustained summer temperatures exceeding 35 °C. However, the increasing frequency of extreme high-temperature events (>40 °C) in recent years has surpassed their evolutionary adaptation threshold, imposing significant heat stress on pregnant sows. This study aims to systematically elucidate the specific mechanisms by which early-pregnancy sows respond to heat stress at physiological, genetic, and metabolic levels, with particular emphasis on the critical role of the liver in heat tolerance. The findings will provide a robust theoretical foundation for the development of heat tolerance-related biomarkers and the breeding of heat-tolerant pig breeds.

## 2. Materials and Methods

### 2.1. Experimental Animals and Environmental Conditions

In this experiment, 20 healthy, 10-month-old, first-parity Rongchang sows with a body weight of approximately 75 kg were randomly selected as samples. After the synchronization of estrus using oral altrenogest (20 mg/day for 18 days), the sows were mated and then randomly assigned to either the heat stress group (RHS-HY) or the thermoneutral group (RHC-HY) on the 6th day post mating. The experiment was conducted in a temperature-controlled chamber. For the RHS-HY group, the ambient temperature was gradually increased at 8:00 to 34 ± 1 °C and gradually decreased at 16:00 to 22 ± 1 °C, with both heating and cooling processes taking approximately 2 h. The RHC-HY group maintained a constant temperature of 22 ± 1 °C throughout the 24 h period. The temperature and humidity in the temperature-controlled room were monitored in real-time over 24 h, and the temperature–humidity index (THI) was calculated using the following formula: THI = (1.8 × T + 32) − [(0.55 − 0.0055 × RH) × (1.8 × T − 26)], where T is the ambient temperature (°C) and RH is the relative humidity (%). Based on daily measurements, the average hourly THI value was calculated. Pregnancy testing was performed 30 days after breeding; only 6 pigs in the heat stress group were confirmed as successfully pregnant. To maintain consistent sample sizes between the two groups for subsequent analyses, 12 individuals (6 per group) were selected for further investigation.

Body surface temperature, rectal temperature, and respiratory rate were manually measured at four time points daily: 9:00, 13:00, 17:00, and 21:00. Rectal temperature was measured using a thermometer. Body surface temperature was assessed using an i-POOK infrared thermometer (Model PK57A, Aiboxiang Technology Co., Ltd., Ning Xia, China), with measurements taken from the ear, shoulder, hip, and tail root. The weighted body surface temperature (Tsk) was calculated using the following formula: Tsk = 0.1 × T_ear + 0.4 × T_shoulder + 0.4 × T_hip + 0.1 × T_tail. Respiratory rate was recorded as breaths per minute. All other husbandry parameters (such as feed, water, and lighting) were kept consistent across treatment conditions to minimize interference from non-heat stress factors. After 45 days of exposure to the set ambient temperature, all pigs were slaughtered for sampling.

### 2.2. Sample Collection and Processing

Immediately after slaughter, 20 mL of blood was collected from each sow. Of this, 5 mL was immediately used for blood gas analysis to ensure data accuracy and reliability. The remaining 15 mL was placed in tubes containing anticoagulants and centrifuged at 3000 r/min for 15 min at 4 °C. The resulting serum was aliquoted and stored at −20 °C until further analysis. Liver tissue samples were collected aseptically using surgical scissors. The samples were immediately placed in liquid nitrogen for transport to the laboratory and subsequently stored at −80 °C for further analysis.

### 2.3. Detection of Hematological, Biochemical, and Endocrine Parameters in Blood

Blood gas analysis: This procedure is used to assess parameters such as oxygen partial pressure (PO_2_), carbon dioxide partial pressure (PCO_2_), pH, blood oxygen saturation, etc. Immediately after sample collection, the i-STAT portable blood gas analyzer (i-STAT Corporation, Princeton, NJ, USA) is employed for testing. Prior to each use, the instrument is calibrated to ensure the accuracy and reliability of the test results.

Blood routine examination: Immediately after blood sample collection, a five-category blood cell analysis is performed. The inspection indicators include the following: neutrophil count (Neu#), lymphocyte absolute value (Lym#), monocyte absolute value (Mon#), eosinophil absolute value (Eos#), basophil absolute value (Bas#), red blood cell count (RBC), white blood cell count (WBC), hemoglobin concentration (HGB), hematocrit (HCT), mean corpuscular volume (MCV), mean corpuscular hemoglobin (MCH), mean corpuscular hemoglobin concentration (MCHC), platelet distribution width coefficient of variation (PDW-CV), platelet distribution width standard deviation (PDW-SD), platelet count (PLT), mean platelet volume (MPV), platelet distribution width (PDW), and plateletcrit (PCT). Biochemical blood tests: Biochemical indicators include albumin (ALB), total protein (TP), globulin (GLB), albumin-to-globulin ratio (A/G), total bilirubin (TBIL), gamma-glutamyl transferase (GGT), aspartate aminotransferase (AST), alanine aminotransferase (ALT), alkaline phosphatase (ALP), and total bile acid (TBA).

An enzyme-linked immunosorbent assay (ELISA) was employed to detect endocrine indicators, inflammatory factors, and antioxidant parameters. Specific endocrine indicators included the following: porcine cortisol (F4551-B), thyroid hormones (T3, F84022-B), thyrotropin (TSH, F4659-B), adrenocorticotropic hormone (ACTH, F8241-B), luteinizing hormone (LH1, F4691-B)(LH2,F811297-B), follicle-stimulating hormone (FSH, F4567-B), glucagon (GC, F84196-B), angiotensin I (Ang-I, F811295-B), insulin (INS, F4542-B), and insulin-like growth factor (IGF-1, F4541-B). Inflammatory factors included pro-inflammatory cytokines IL-6 (F4570-96T), TNF-α (F4535-96T), and IL-1β (F4574-96T) and the anti-inflammatory cytokine IL-10 (F4577-96T), TGF-α (F811234-96T), and lactic dehydrogenase L-LDH (LDH-2-Y). Antioxidant parameters included total antioxidant capacity (T-AOC, FRAP-2-G), malondialdehyde (MDA, MDA-2-Y), and superoxide dismutase (SOD, SOD-2-W). All assays were conducted using kits from FANKEW (Shanghai, China) strictly according to the manufacturer’s instructions to ensure the reliability and reproducibility of the results.

### 2.4. Histopathological and Immunofluorescence Analysis of Liver

Liver tissue was fixed in 4% paraformaldehyde to ensure optimal fixation. The fixed tissues were subsequently dehydrated, paraffin-embedded, and sectioned. After sectioning, the slides underwent dewaxing and hematoxylin–eosin (H&E) staining. The stained sections were re-dehydrated and sealed with neutral gum to prepare sample blocks for microscopic and histopathological examination.

Immunofluorescence staining was employed to evaluate the effects of heat stress on apoptosis, stress response, and immune response in liver cells. The specific protocol is as follows: First, paraffin sections were sequentially placed in an environmentally friendly dewaxing solution and anhydrous ethanol, dewaxed to water, and washed with distilled water. The sections were then subjected to antigen retrieval in EDTA buffer using a microwave oven and subsequently washed three times with PBS buffer for 5 min each. Circles were drawn around the sections using an immunohistochemistry pen to prevent antibody diffusion. Sections were blocked with 1% bovine serum albumin (BSA) solution for 30 min. Liver injury scores were assessed according to the following criteria: pathological changes that slightly exceeded the normal range were classified as very mild; lesions that were detectable but not severe were classified as mild; lesions that were clearly visible and potentially progressing to a more serious state were classified as moderate; and lesions that were highly severe, such as those extensively affecting the entire tissue or organ, were classified as severe.

All primary antibodies, including Bax (GB114122), Bcl-2 (GB113375), HSP70 (GB15241), HSP90 (GB12284), CD3 (GB12014), and CD8 (GB12068), were purchased from Servicebio (Wuhan, China) and incubated overnight at 4 °C. The antibody dilution standards for immunofluorescence were prepared according to the manufacturer’s instructions as follows: BAX at 1:400, BCL-2 at 1:500, CD8 at 1:500, HSP70 at 1:500, HSP90 at 1:500, and CD3 at 1:1000. The next day, sections were washed three times with PBS for 5 min each, followed by incubation with the corresponding secondary antibodies at room temperature for 50 min. Nuclei were counterstained with DAPI for 10 min in the dark, and spontaneous fluorescence quenching was performed. Finally, sections were mounted with an anti-fade mounting medium and examined under a fluorescence microscope to record the immunofluorescence staining results. Both the experimental and control groups for analyses were established with three biological replicates and three technical replicates each.

### 2.5. Transcriptome, Proteome, and Metabolome Analysis

RNA-seq, proteomics, and non-targeted metabolomics were performed on liver tissues from the RHS-HY group (*n* = 3: RHS-H-li1, RHS-H-li2, and RHS-H-li3) and the RHC-HY group (*n* = 3: RHC-H-li1, RHC-H-li2, and RHC-H-li3).

Transcriptomic analysis: RNA was extracted from liver tissues and subjected to stringent quality control using an Agilent 2100 Bioanalyzer (Agilent Technologies, Palo Alto, CA, USA). Subsequently, RNA libraries were constructed and sequenced using high-throughput sequencing technology. The transcriptome dataset comprises 6G of sequencing data, and the reference genome utilized is the Ssrofa11.1 version from NCBI. The raw sequencing data underwent quality control, filtering, error rate assessment, and GC content distribution evaluation to generate clean reads for downstream analysis. For quality control of the sequencing data in this project, the error rate must not exceed 0.2%, with Q20 values required to be greater than 90% and Q30 values exceeding 85%. Additionally, the GC content should ideally range between 40% and 60%. FPKM, which accounts for both sequencing depth and gene length effects on read counts, remains the most widely adopted method for estimating gene expression levels. Differential expression analysis was performed using the DESeq2 R package (1.42.0, Michael Love, Chapel Hill, NC, USA). The mRNA sequencing data have been deposited in the SRA database under BioProject accession number PRJNA1209205. Clean reads were then aligned to the reference genome using HISAT2 software (2.2.1, Steven Salzberg et al., Baltimore, MD, USA) for rapid and precise mapping. Based on the alignment results, the number of reads covering each gene (including newly predicted genes) was counted, and the expression levels (fragments per kilobase of transcript per million mapped reads, FPKM) of all genes in each sample were calculated. Differential gene expression was identified using the criteria of |log2(FoldChange)| ≥ 1 and adjusted *p*-value (*p*adj) ≤ 0.05. Finally, GO functional enrichment analysis and KEGG pathway enrichment analysis of the differentially expressed genes were performed using clusterProfiler software (3.10.1, Yu G., Guangzhou, China).

Proteomic analysis: Liver tissues were retrieved from a −80 °C freezer and ground into powder at low temperature. The powdered samples were immediately transferred to centrifuge tubes pre-cooled with liquid nitrogen. An appropriate amount of SDT lysis buffer (containing 100 mM NaCl) and DTT (at a volume ratio of 1/100) was added, followed by thorough mixing through vortexing. The samples were then sonicated in an ice-water bath for 5 min to ensure complete cell lysis. Subsequently, the samples were incubated at 95 °C for 8–15 min, cooled on ice for 2 min, and centrifuged at 4 °C and 12,000× *g* for 15 min. The supernatant was collected and treated with an adequate amount of IAM solution under dark conditions for 1 h. Four volumes of pre-chilled acetone (−20 °C) were added to precipitate proteins at −20 °C for at least 2 h. The samples were then centrifuged at 4 °C and 12,000× *g* for 15 min, and the resulting precipitates were collected. The precipitates were re-suspended in 1 mL of pre-chilled acetone (−20 °C), washed, dried, and finally dissolved in dissolved buffer (DB buffer).

The extracted proteomes were sent to Novogene Bio (Tianjin, China) for protein detection using the Vanquish™ Neo UHPLC-Astral DIA method (Thermo Fisher Scientific, Waltham, MA, USA). Raw files were searched and analyzed using the DIA-NN software (0.97.2, Markus Ralser, Berlin, Germany) against the Uniprot protein database. The proteomics data have been deposited in the ProteomeXchange via the PRIDE database under Project Accession Number PXD059676. The false discovery rate (FDR) for peptides was not controlled, whereas the protein-level FDR was set at 0.01. The fold change (FC) was calculated as the ratio of the mean relative quantification values of the experimental group to those of the control group. For *p*-value calculation, zero values were excluded prior to conducting a two-sample *t*-test. Additionally, an F-test for the homogeneity of variance was performed beforehand to assess whether there was a significant difference in variances between the two groups.

The functional annotation of the identified proteins was performed using Interproscan software (v105.0, EBI, Cambridge, UK), which included GO and IPR annotations based on Pfam, PRINTS, ProDom, SMART, ProSite, and PANTHER databases. COG and KEGG analyses were conducted to classify the proteins into functional families and pathways. Differential protein expression was further analyzed using volcano plots, cluster heatmaps, and pathway enrichment analyses of GO, IPR, and KEGG. Additionally, STRING DB software (v12.0, USA) was used to predict potential protein–protein interactions (http://string-db.org/, accessed on 16 December 2024).

Non-targeted metabolomics analysis: The non-targeted metabolomics study of liver tissue was conducted using liquid chromatography-mass spectrometry (LC-MS) technology. The experimental process included metabolite extraction from liver tissue samples, LC-MS/MS detection, and data analysis. The extraction procedure for liver tissue metabolites is as follows: Homogenize 100 mg of tissue samples in liquid nitrogen and transfer them to EP tubes. Add 500 μL of an 80% methanol aqueous solution. Vortex thoroughly and incubate on ice for 5 min. Centrifuge at 15,000× *g* for 20 min at 4 °C. Collect an appropriate volume of the supernatant and dilute it with mass spectrometry-grade water to adjust the methanol content to 53%. Perform a second centrifugation at 15,000× *g* for 20 min at 4 °C, and collect the supernatant for LC-MS analysis. The database information used for the spectral library search includes the mzCloud database, mzVault database, and Masslist database. Raw files obtained from mass spectrometry were imported into Compound Discoverer 3.3 software for spectral processing and database searching to obtain qualitative and quantitative results of metabolites. Quality control measures were implemented to ensure the accuracy and reliability of the data. Differential metabolites were identified based on criteria of variable importance in projection, VIP > 1.0, FC > 1.2 or FC < 0.833, and *p*-value < 0.05. Subsequently, multivariate statistical analyses, including principal component analysis (PCA) and partial least squares discriminant analysis (PLS-DA), were performed to reveal differences in metabolic patterns among different groups. Hierarchical clustering (HCA) and metabolite correlation analysis were used to elucidate relationships between samples and among metabolites. Finally, functional and classification annotations of the identified metabolites were conducted, and their biological significance was interpreted through pathway analysis.

### 2.6. Correlation Analysis of Proteomics, Transcriptomics, and Metabolomics

The expression levels of genes, proteins, and metabolites in liver tissues were comprehensively analyzed. All identified differentially expressed genes, proteins, and metabolites were mapped to the KEGG pathway database to obtain their common pathway information. This allowed us to identify the major biochemical pathways and signaling pathways commonly involved by differential metabolites, proteins, and genes. Based on these enrichment results, we performed KEGG Pathview visualization analysis for metabolic pathways co-enriched by metabolomics, proteomics, and transcriptomics data. In the KEGG pathway maps, circles represent metabolites, with blue circles indicating downregulated differential metabolites and yellow circles indicating upregulated differential metabolites. The left half of each box represents proteins, while the right half represents genes; green shading indicates downregulated expression, and red shading indicates upregulated expression.

### 2.7. Data Analysis

A one-way analysis of variance (ANOVA) and independent-samples *t*-tests were conducted to analyze the data using SPSS 22.0 (IBM SPSS Statistics, Chicago, IL, USA), with significance set at *p* < 0.05. All results are expressed as mean ± standard deviation (SD). Radar plots, UpSet plots, and heatmaps were generated using R packages (4.2.2, Ross Ihaka and Robert Gentleman, Auckland, New Zealand). The figures were created using Adobe Illustrator CC (2017). Each experimental group included at least three biological replicates. Statistical significance was defined as *p* < 0.05, with the following notations: *, *p* < 0.05, **, *p* < 0.01, and ***, *p* < 0.001.

## 3. Results

### 3.1. Environmental Control, Body Temperature, and Respiratory Rate

**Figure 1:**
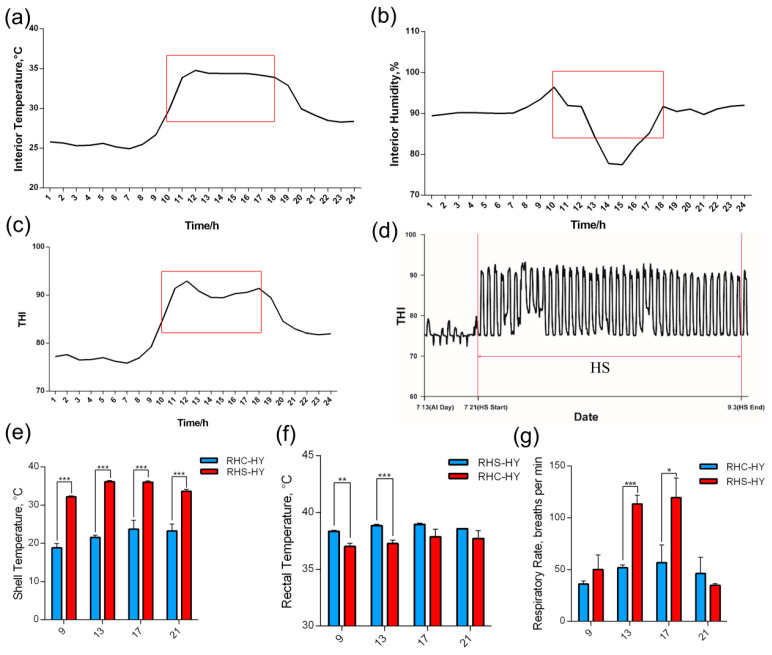
Environmental control and monitoring of body temperature and respiratory rate in sows during early gestation under HS conditions. (**a**) Daily average indoor ambient temperature, (**b**) daily average interior humidity, (**c**) daily average temperature–humidity index (THI) value, (**d**) THI variation over time, (**e**) surface body temperature, (**f**) rectal temperature, and (**g**) respiratory rate. Note: The red box highlights the specific time intervals designated for heat stress exposure. *, *p* < 0.05; **, *p* < 0.01; and ***, *p* < 0.001.

We evaluated the hourly average values of environmental temperature and humidity in the temperature-controlled room under heat stress (HS) conditions. From 9:00 to 17:00, the indoor temperature was maintained within the range of 32–35 °C (Figure 1a). During the HS period, indoor humidity reached 85–92% (Figure 1b), and the temperature–humidity index (THI) value remained at approximately 90 (Figure 1c,d), fulfilling the experimental criteria for heat stress conditions. To determine whether heat stress occurred, we measured the body surface and rectal temperatures of the sows. The results demonstrated that, compared with the RHC-HY group, the body surface temperature of sows in the RHS-HY group under HS conditions was significantly elevated at all four time points (9:00, 13:00, 17:00, and 21:00) (*p* < 0.001) (Figure 1e). Additionally, the rectal temperature was significantly increased at 9:00 (*p* < 0.01) and 13:00 (*p* < 0.001) (Figure 1f). Furthermore, the respiratory rate of sows in the RHS-HY group was significantly higher at 13:00 (*p* < 0.001) and 17:00 (*p* < 0.05) (Figure 1g). At these two time points, the respiratory rates of sows in the RHS-HY group were 61.62 bpm and 62.76 bpm higher than those in the RHC-HY group, respectively. Based on the aforementioned findings, under the established environmental conditions, the sows exhibited physiological responses indicative of heat resistance.

### 3.2. Changes in Hematological Parameters, Blood Acid–Base Balance, and Oxygen Metabolism Under HS

Compared with the RHC-HY group, the RHS-HY group exhibited significantly lower Bas levels and proportions (*p* < 0.05), whereas red blood cell count (RBC) (*p* < 0.01), HGB (*p* < 0.001), and HCT (*p* < 0.001) were significantly higher. Only the RDW-CV was significantly reduced (*p* < 0.05). No significant differences were observed in other indicators (Table 1). Additionally, compared with the RHC-HY group, the partial pressure of PO_2_ in the blood of the RHS-HY group was significantly decreased (*p* < 0.001), while TCO_2_, Na, and Hb levels were significantly increased (*p* < 0.05). Other indices showed no significant changes (Table 2).

### 3.3. Analysis of Serum Hormone Levels and Liver Function Indices

**Figure 2:**
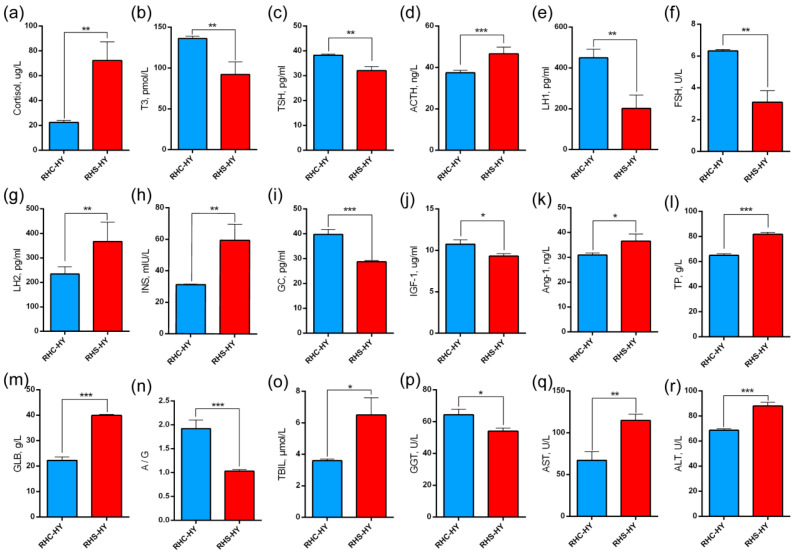
Analysis of serum hormone levels and liver function indexes. (**a**) The level of cortisol, (**b**) the level of T3, (**c**) the level of thyroid stimulating hormone (TSH), (**d**) the level of adreno-cortico-tropic-hormone (ACTH), (**e**) the level of luteinizing hormone 1 (LH1), (**f**) the level of follicle-stimulating hormone (FSH), (**g**) the level of luteinizing hormone 2 (LH2), (**h**) the level of insulin (INS), (**i**) the level of glucagon (GC), (**j**) the level of insulin-like growth factor-1 (IGF-1), (**k**) the level of angiopoietin-1 (Ang-1), (**l**) the level of total protein (TP), (**m**) the level of globulin (GLB), (**n**) albumin/globulin (ALB/GLB), (**o**) the level of total bilirubin (TBIL), (**p**) the level of gamma-glutamyl transpeptidase (GGT), (**q**) the level of aspartate aminotransferase (AST), and (**r**) the level of alanine aminotransferase (ALT). Note: Bars represent geometric means ± SD. *, *p* < 0.05; **, *p*< 0.01; and ***, *p* < 0.001 using a one-way analysis of variance (ANOVA) followed by Tukey’s test.

To further evaluate the physiological responses of pregnant sows under heat stress conditions, we analyzed serum hormone levels and liver function indicators. Compared with the RHC-HY group, the RHS-HY group exhibited significantly elevated cortisol levels (72.37 ± 14.90 vs. 22.51 ± 1.55 μg/L) (*p* < 0.001) (Figure 2a). Thyroid function-related indicators showed significant changes, with T3 and TSH levels significantly decreasing (92.19 ± 15.47 vs. 136.20 ± 2.87 pmol/L and 34.07 ± 4.64 vs. 38.28 ± 0.48 pg/mL, respectively) (*p* < 0.01) (Figure 2b,c). Additionally, adrenal function was significantly altered, as evidenced by a significant increase in ACTH expression (46.66 ± 3.17 vs. 37.44 ± 1.18 ng/L) (*p* < 0.001) (Figure 2d). Reproductive hormone levels also changed: LH1 and FSH levels were significantly reduced (202.31 ± 65.32 vs. 449.79 ± 42.05 pg/mL and 3.09 ± 0.73 vs. 6.32 ± 0.09 U/L) (*p* < 0.01) (Figure 2e,f), while LH2 levels were significantly increased (366.15 ± 80.03 vs. 234.23 ± 28.94 pg/mL) (*p* < 0.01) (Figure 2g). Metabolism-related indicators revealed that INS levels in the RHS-HY group were significantly higher (*p* < 0.01) (59.51 ± 10.25 vs. 31.23 ± 0.31 mU/L) (Figure 2h), whereas GC (28.74 ± 0.50 vs. 39.78 ± 1.99 pg/mL) (*p* < 0.001) and IGF-1 (9.93 ± 1.20 vs. 10.74 ± 0.55 μg/mL) (*p* < 0.05) levels were significantly lower (Figure 2i,j). Furthermore, the expression of Ang-I was significantly upregulated (36.58 ± 2.20 vs. 30.97 ± 0.84 ng/L) (*p* < 0.05) (Figure 2k).

**Figure 3:**
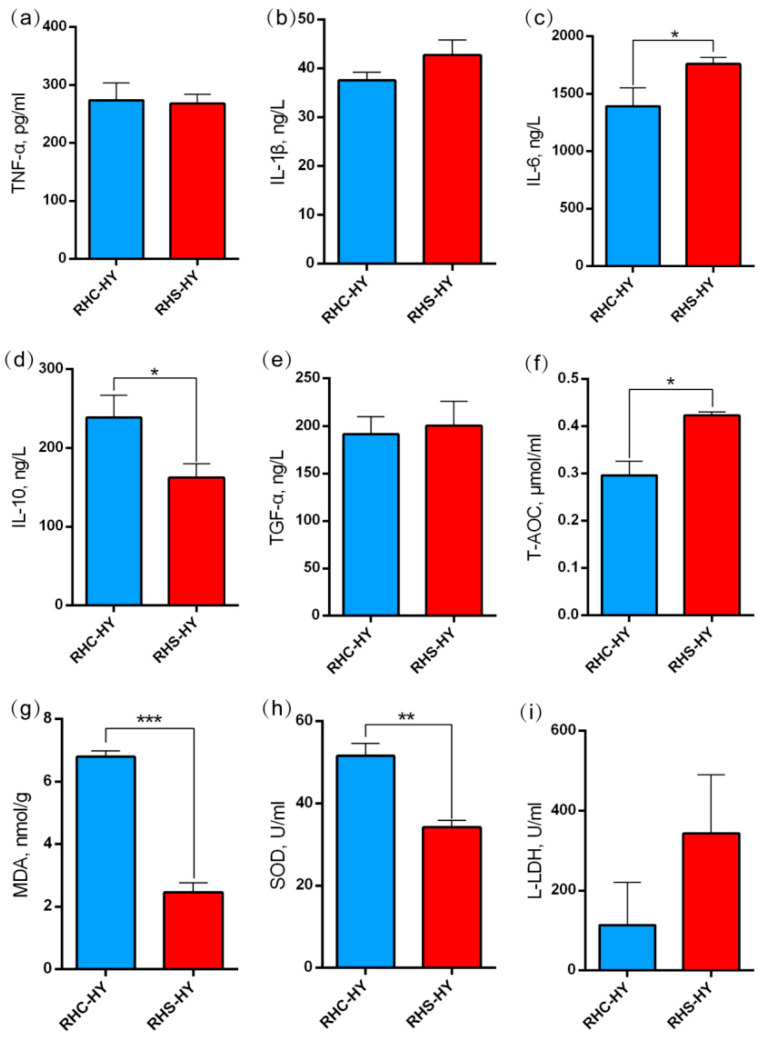
Analysis of liver inflammation and antioxidant capacity. (**a**) The level of tumor necrosis factor-α (TNF-α); (**b**) the level of interleukin-1β (IL-1β); (**c**) the level of interleukin-6 (IL-6); (**d**) the level of interleukin-10 (IL-10); (**e**) the level of transforming growth factor-α (TGF-α); (**f**) the level of total antioxidant capacity (T-AOC); (**g**) the level of malondialdehyde (MDA); (**h**) the level of superoxide dismutase (SOD); and (**i**) the level of lactate dehydrogenase L (L-LDH). Note: Bars represent geometric means ± SD. *, *p* < 0.05; **, *p* < 0.01; and ***, *p* < 0.001 using a one-way analysis of variance (ANOVA) followed by Tukey’s test.

Simultaneously, the analysis of liver function indicators revealed that compared with the RHC-HY group, the levels of TP, GLB, TBIL, AST, and ALT in the RHS-HY group were significantly elevated (*p* < 0.05). Notably, the levels of TBIL and AST exceeded the reference ranges: TBIL was 6.50 ± 1.10 μmol/L (reference range: 0.0–5.0 μmol/L) and AST was 114.67 ± 7.51 U/L (reference range: 16–65 U/L) (Figure 2l–r). Additionally, we analyzed the A/G ratio and found it to be significantly reduced (*p* < 0.001) (Figure 2n), indicating that heat stress (HS) caused hepatocyte damage, thereby affecting liver metabolism, detoxification, and anti-inflammatory functions. Compared with the RHC-HY group, the pro-inflammatory cytokine IL-6 in the RHS-HY group was significantly increased (*p* < 0.05) (Figure 3c), while the levels of TNF-α and IL-1β showed no significant differences (Figure 3a,b). The expression level of the anti-inflammatory cytokine IL-10 was significantly decreased (*p* < 0.05) (Figure 3d), further suggesting an imbalance in inflammatory responses. To further evaluate antioxidant capacity, we conducted additional tests. The results indicated that compared with the RHC-HY group, total antioxidant capacity T-AOC in the RHS-HY group was significantly increased (*p* < 0.05) (Figure 3f), whereas MDA and SOD levels were significantly decreased (*p* < 0.05) (Figure 3g,h). L-LDH showed an increasing trend (Figure 3i). The above findings comprehensively indicate that heat stress impairs liver function and increases inflammatory levels in pregnant sows. Additionally, the results suggest that the RHS-HY group exhibited enhanced overall antioxidant defense system activity, with the antioxidant system effectively neutralizing free radicals.

### 3.4. Analysis of Liver Histological Features and Labeled Proteins

**Figure 4:**
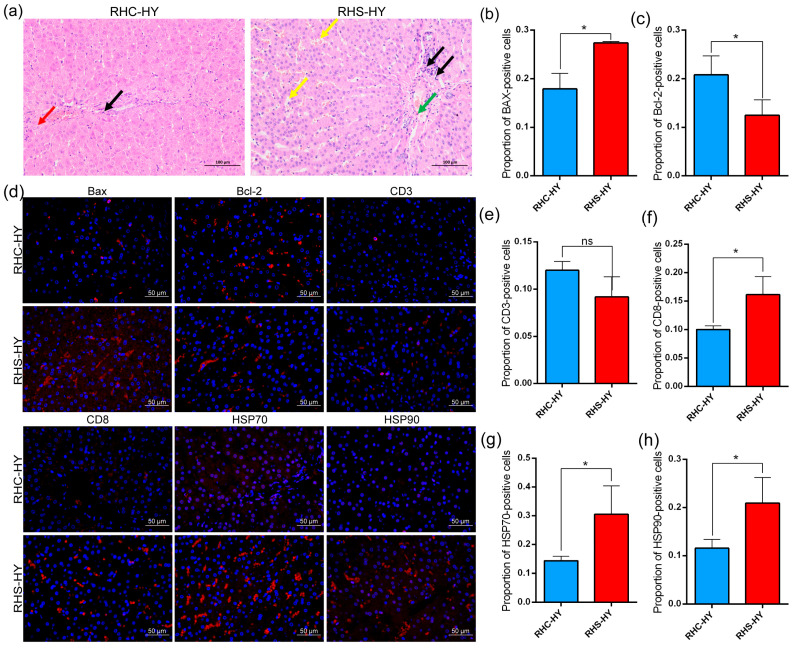
Analysis of liver histological characteristics. (**a**) Histopathological analysis of liver sections, (**b**) proportion of Bax-positive cells, (**c**) proportion of Bcl-2-positive cells, (**d**) immunofluorescence images showing the proportions of Bax, BCL-2, CD3, CD8, HSP70, and HSP90 positive cells in liver tissue, (**e**) proportion of CD3-positive cells, (**f**) proportion of CD8-positive cells, (**g**) proportion of HSP70-positive cells, and (**h**) proportion of HSP90-positive cells. Note: number of lymphocytes (indicated by black arrows), sporadic hepatocyte necrosis (green arrows), a mild dilation of hepatic sinusoids (yellow arrows), hydropic degeneration of hepatocytes surrounding the central vein (red arrows); *, *p* < 0.05; NS stands for no significant difference.

Given the abnormal liver function observed in the blood, we analyzed pathological changes in liver tissue under HS. In the RHS-HY group, a small number of lymphocytes (indicated by black arrows) were observed around portal areas, accompanied by sporadic hepatocyte necrosis (green arrows), a mild dilation of hepatic sinusoids (yellow arrows), and disorganized hepatic cords with widened spacing (Figure 4a). No significant necrosis or abnormalities were detected in the liver tissues of the RHC-HY group. Thus, compared to the RHC-HY group, the RHS-HY group exhibited marked liver tissue damage. Further analysis confirmed these findings through apoptotic activity assessment. Immunofluorescence assays revealed that, compared with the RHC-HY group, the pro-apoptotic protein Bax was significantly elevated (*p* < 0.05) (Figure 4b), while anti-apoptotic protein Bcl-2 levels were significantly reduced (*p* < 0.05) (Figure 4c), indicating enhanced apoptotic activity. Additionally, no significant difference was observed in CD3-positive cells (Figure 4e); however, CD8 expression was significantly increased (*p* < 0.05) (Figure 4f), suggesting the activation of cytotoxic T lymphocytes (CTLs), which may contribute to further hepatocyte damage. Moreover, heat shock proteins HSP70 and HSP90 in hepatocytes were significantly upregulated (*p* < 0.05) (Figure 4g,h), likely as a protective response to protein damage and cellular stress induced by heat stress.

### 3.5. Transcriptome Analysis of Sows During Early Pregnancy Under HS

**Figure 5:**
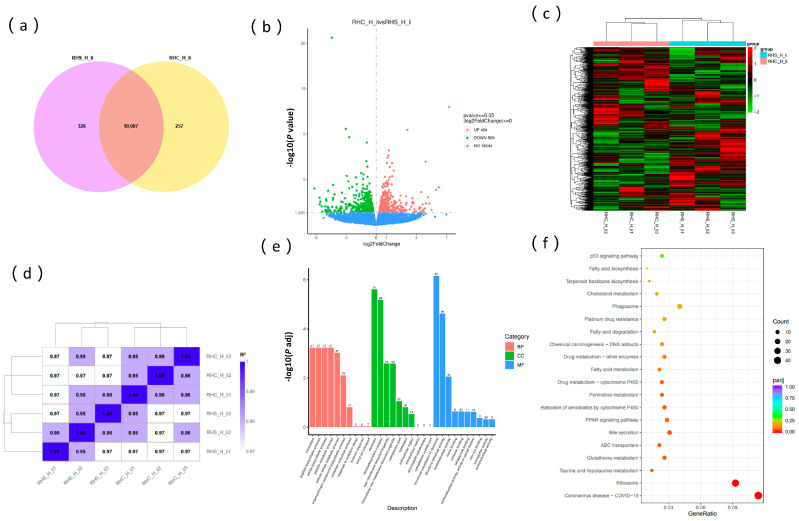
Transcriptome analysis of differentially expressed genes (DEGs) in the liver of early-pregnancy Rongchang sows. (**a**) Violin plots of the different gene expressions; (**b**) Venn diagram of significant DEGs between RHS-HY and RHC-HY; (**c**) statistical representation of significant DEGs between RHS-HY and RHC-HY; (**d**) cluster heatmap of samples; (**e**) heatmap of the correlation between different samples; (**f**) GO enrichment of DGEs between RHS-HY and RHC-HY groups; and (**g**) KEGG enrichment of DGEs between RHS-HY and RHC-HY groups.

The RHS-HY and RHC-HY groups generated 135,774,834 and 153,692,812 clean reads, respectively (Table 2), with a total base count of 43.41 Gb (≥6.3 Gb per sample). Quality assessment revealed Q20 > 98.58%, Q30 > 95.94%, and GC content between 49.84% and 50.43% (Table S1). Combined with qualified FPKM values, these metrics confirm the high quality of transcriptome sequencing data (Figure 5a). Comparative transcriptomic analysis between the RHS-HY and RHC-HY groups identified 10,007 commonly expressed genes, with 326 upregulated and 257 downregulated genes in the RHS-HY group relative to controls (Figure 5b). Applying a significance threshold (*p* < 0.05), we detected 992 differentially expressed genes (DEGs), comprising 508 upregulated and 484 downregulated genes (Figure 5c). The hierarchical clustering of DEGs revealed a clear segregation of the two experimental groups (Figure 5d), supported by high intra-group consistency (Pearson correlation coefficients > 0.97; Figure 5e). The functional annotation of DEGs through GO enrichment analysis assigned 13,570 terms, predominantly in molecular functions (49.9%), followed by biological processes (31%) and cellular components (19%), indicating their primary regulatory roles in hepatic molecular functions such as oxidoreductase activity, peptide biosynthesis, and amide metabolism (Figure 5f). KEGG pathway analysis further demonstrated significant enrichment in taurine/hypotaurine metabolism, glutathione metabolism, bile secretion, PPAR signaling, and pyrimidine metabolism (Figure 5g).

### 3.6. Overview of the Proteome Data and Correlation Analysis with Transcriptome

**Figure 6:**
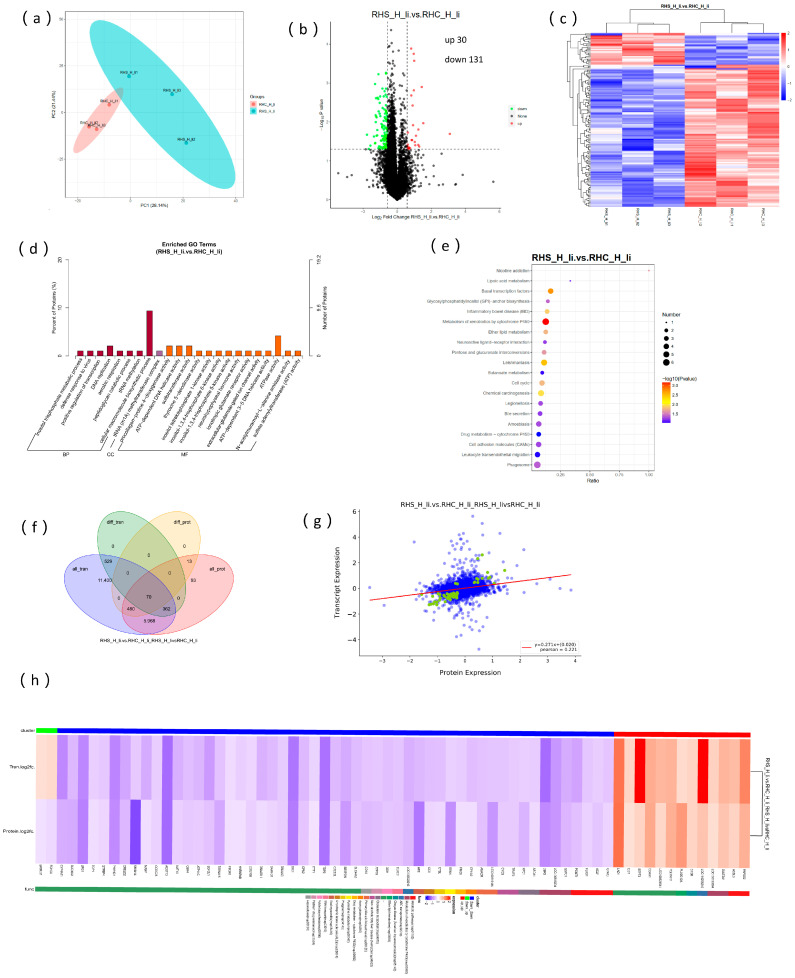
Proteome and transcriptome association analysis. (**a**) PCA of protein profiles in the RHC-HY and RHS-HY groups; (**b**) differential protein expression analysis between the RHC-HY and RHS-HY groups; (**c**) hierarchical clustering of differentially expressed proteins; (**d**) gene ontology (GO) enrichment analysis of differentially expressed proteins; (**e**) KEGG pathway analysis of differentially expressed proteins; (**f**) Venn diagram illustrating co-expression patterns of genes and proteins; (**g**) correlation analysis of differential gene and protein expression levels; and (**h**) KEGG pathway analysis of co-expressed genes and proteins.

The results of the protein control are shown in Figure S1. The principal component analysis (PCA) of protein expression in Figure 6a revealed that the RHS-HY group and RHC-HY group were clearly separated into two distinct clusters. Using a fold change (FC) threshold of ≥1.2 and *p*-value ≤ 0.05, we identified upregulated proteins; using an FC threshold of ≤0.83 and *p*-value ≤ 0.05, we identified downregulated proteins. A total of 161 differentially expressed proteins (DEPs) were detected between the RHS-HY and RHC-HY groups, with 30 proteins upregulated and 131 proteins downregulated (Figure 6b). Among these proteins, carbonyl reductase [NADPH] 1-like exhibited an 8-fold upregulation in the RHS-HY group. Hierarchical clustering analysis further confirmed that the RHS-HY and RHC-HY groups could be distinctly divided into two clusters (Figure 6c). The GO enrichment analysis of these DEPs indicated that they were primarily enriched in biological processes and molecular functions, particularly those related to enzyme activity and energy metabolism. Key terms included procollagen-proline 4-dioxygenase activity, ATP-dependent DNA helicase activity, sulfotransferase activity, ATP-dependent 3′-5′ DNA helicase activity, and ATPase activity (Figure 6d). KEGG pathway analysis showed significant enrichment in pathways such as xenobiotic metabolism by cytochrome P450 and basal transcription factors (Figure 6e). Correlation analysis between the transcriptome and proteome revealed that 70 genes and proteins were co-expressed between the RHS-HY and RHC-HY groups (Figure 6f). KEGG pathway analysis indicated that the expression patterns of these co-expressed genes and proteins were largely consistent (Figure 6g). These genes and proteins were primarily enriched in pathways such as metabolic pathways, cholesterol metabolism, primary bile acid biosynthesis, pyrimidine metabolism, and ether lipid metabolism (Figure 6h).

### 3.7. Analysis of Liver Metabolic Characteristics and the Association with Transcriptome and Proteome

**Figure 7:**
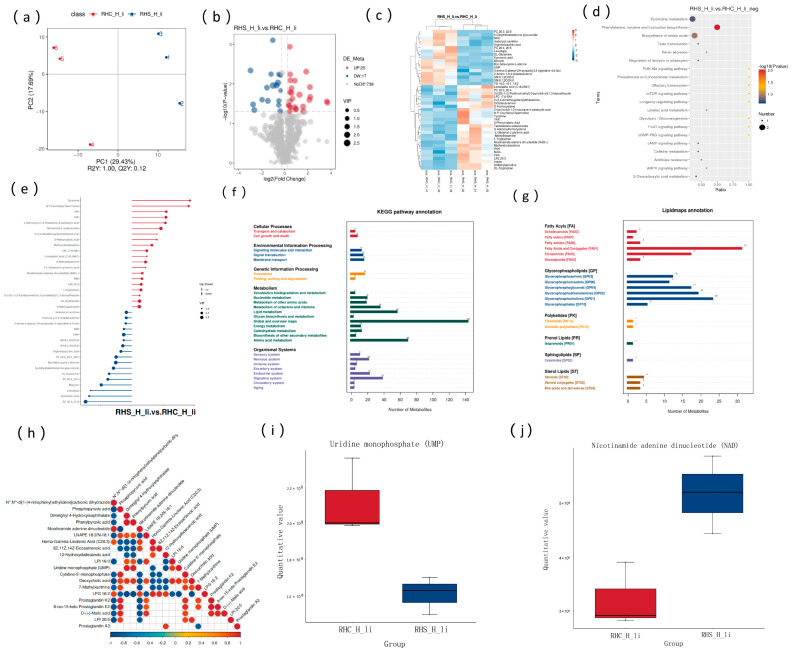
Liver metabolic characteristics and their association with transcriptome and proteome. (**a**) Partial least squares discriminant analysis (PLS-DA) of metabolites (pos); (**b**) differential expression analysis of metabolites; (**c**) hierarchical clustering of differentially expressed metabolites; (**d**) enrichment analysis of differentially expressed metabolites; (**e**) differentially expressed metabolites; (**f**) KEGG pathway annotation of differentially expressed metabolites; (**g**) annotation of lipid metabolites; (**h**) correlation analysis of differentially expressed metabolites; (**i**) expression levels of UMP in the RHS-HY and RHC-HY groups; and (**j**) expression levels of NAD in the RHS-HY and RHC-HY groups.

The PLS-DA analysis of metabolites revealed that the experimental models for RHS-HY and RHC-HY groups were well established (Figure 7a). In the positive ion mode, 21 differential metabolites were identified between the RHS-HY and RHC-HY groups, with fourteen downregulated and seven upregulated. In the negative ion mode, a total of 42 differential metabolites were detected, including 25 upregulated and 17 downregulated (Figure 7b). Cluster analysis showed that these differential metabolites clearly clustered into two distinct groups according to the experimental conditions, with high sample consistency (Figure 7c). KEGG pathway analysis indicated that the differential metabolites were primarily enriched in pathways such as phenylalanine, tyrosine, and tryptophan biosynthesis (Figure 7d). Functional annotation revealed that upregulated metabolites included prostaglandin A3, hydroxydodecanoic acid, nicotinamide adenine dinucleotide (NAD), and lysophosphatidylglycerol (LPCs), among others. Downregulated metabolites included uracil nucleotides, lysophosphatidylserine (16:0), adenosine 5′-monophosphate (AMP), D-(+)-malic acid, etc. (Figure 7e). KEGG enrichment analysis further showed that the main pathways involved were Metabolism, Organismal Systems, and Environmental Information Processing (Figure 7f). Lipid metabolites were primarily categorized into glycerophospholipids, fatty acids, and sterol lipids (Figure 7g). The correlation analysis of key differential metabolites revealed that NAD was positively correlated with N″,N‴-di [1-(4-nitrophenyl)ethylidene]carbonic dihydrazide and negatively correlated with phosphopyruvic acid. Uridine monophosphate (UMP) was positively correlated with dimethyl 4-hydroxyisophthalate and phenylpyruvic acid but negatively correlated with homo-gamma-linolenic acid (C20:3) and 12-hydroxydodecanoic acid (Figure 7h). Notably, in the RHS-HY group, NAD expression was significantly upregulated (Figure 7i), while UMP expression was significantly downregulated (Figure 7j).

### 3.8. Focus on Oxidation Resistance Through Multi-Omics Analysis

**Figure 8:**
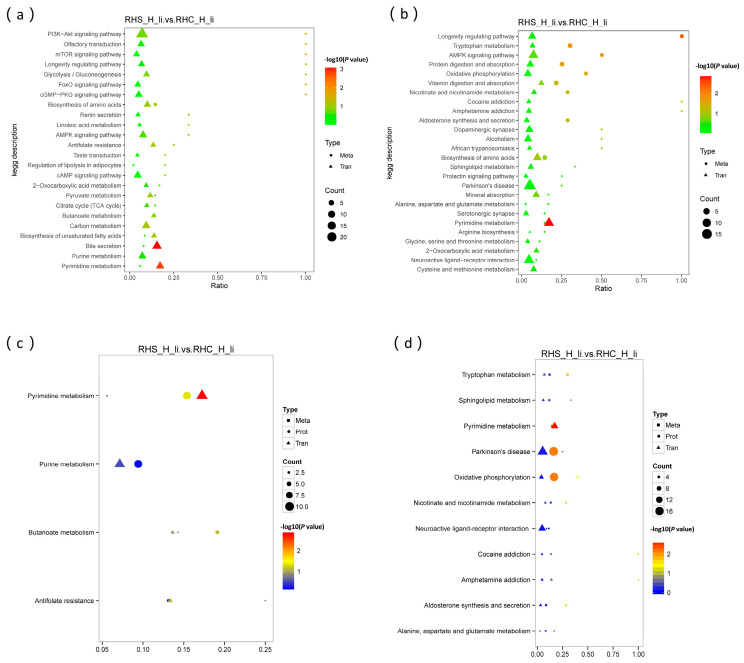
Association analysis of differential metabolites, genes, and proteins. (**a**) KEGG pathway enrichment analysis of the association between differential metabolites and genes (neg); (**b**) KEGG pathway enrichment analysis of the association between differential metabolites and genes (pos); (**c**) KEGG pathway enrichment analysis of the correlation among differential metabolites, genes, and proteins (neg); (**d**) KEGG pathway enrichment analysis of the correlation among differential metabolites, genes, and proteins (pos).

By correlating differential metabolites with gene expression, it was found that in the negative ion mode, significant enrichment occurred primarily in pyrimidine metabolism and bile secretion pathways. The key metabolite involved is AMP, which exhibits significant antioxidant properties (Figure 8a). In the positive ion mode, enrichment was mainly observed in pyrimidine metabolism, the AMPK signaling pathway, protein digestion and absorption, oxidative phosphorylation, and vitamin digestion and absorption pathways (Figure 8b). The primary metabolite involved here is NAD, which also plays a crucial role in antioxidant defense (Table S2). The results of multi-omics association analysis revealed that in the negative ion mode, KEGG pathways were mainly enriched in Pyrimidine metabolism and Oxidative phosphorylation, with UMP being the key metabolite involved (Figure 8c). In the positive ion mode, KEGG pathways were significantly enriched in Pyrimidine metabolism, Oxidative phosphorylation, and Tryptophan metabolism (Figure 8d). The main metabolites identified were UMP, NAD, kynurenic acid, indole, and L-tryptophan (Table S3). These metabolites are known to enhance the body’s anti-stress capabilities. In summary, the combined transcriptome, proteome, and metabolome multi-omics analysis revealed that the liver of sows in early pregnancy under HS conditions exhibits important antioxidant functions. This is achieved through the regulation of key metabolites such as adenosine 5′-monophosphate (AMP), NAD+, and UMP via multiple metabolic pathways.

## 4. Discussion

### 4.1. Establish the Heat Stress Environment for Early Gestation Sows

In this study, a heat stress environment was established by precisely controlling temperature and humidity to evaluate the biological effects and genetic regulatory mechanisms of heat stress (HS) on sows during early gestation. Typically, the optimal temperature range for pregnant sows is 10–27 °C. However, pregnancy can be divided into three stages: early (0–52 days), mid (58.5 ± 5.7 days), and late (104.7 ± 2.8 days). Each stage has distinct thermal requirements; for instance, sows in late gestation prefer temperatures 0.8 °C lower than those in mid-gestation [22]. Therefore, in this study, the RHC-HY group was maintained at a thermoneutral temperature of 22 ± 1 °C, while the RHS-HY group underwent HS treatment from day 6 to day 52 post breeding. Based on the findings of Betty R. McCon et al., the ambient temperature for the RHS-HY group was set at 34 ± 1 °C for 8 h daily, which represents severe heat stress conditions [17]. The temperature–humidity index (THI) plays a crucial role in the physiological responses and litter size of sows during early pregnancy. It is generally accepted that a THI range of 60–70 is optimal for pregnant sows. Specifically, THI levels between 75 and 78 indicate mild heat stress, levels between 79 and 83 indicate moderate heat stress, and values above 84 indicate severe heat stress [7,23]. In this study, the THI value for the RHS-HY group was maintained at approximately 90, indicating a state of severe heat stress both on individual days and throughout the entire experimental period. Under HS, the basal metabolic rate and respiratory rate of the pigs were significantly elevated to enhance heat dissipation through increased surface cooling and respiratory panting. These findings are consistent with previous studies [24]. Additionally, in this study, we observed that the rectal temperature of sows during early gestation increased significantly during the initial phase of HS and stabilized in the later phase. This stabilization may be attributed to reduced activity levels, indicating a certain degree of heat resistance developed by the sows [25], these findings suggest that we successfully established a heat stress model for sows in early gestation.

### 4.2. Blood Oxygen Levels Decrease, Leading to Inflammation

We further evaluated the physiological responses of sows in early pregnancy under heat stress (HS). Blood biochemical analysis revealed that, under HS conditions, PO_2_ levels in the blood of sows in the RHS-HY group were significantly reduced, while TCO_2_ levels were significantly elevated. This suggests that the increased oxygen demand may have led to a decrease in systemic oxygen content [26], indicating that these sows were in a state of hypoxia. Consequently, this hypoxic condition resulted in lactic acid accumulation, which adversely affected their overall health and placental function [27]. Studies have demonstrated that exposure to hypoxic conditions during pregnancy can lead to reduced fetal weight, developmental restrictions, and diminished adaptive capacity [28,29]. A significant reduction in RDW-CV levels enhances the oxygen-carrying capacity of the blood. The early gestation period is critical for embryo implantation and placental development, which requires an increased number of red blood cells to support oxygen and nutrient delivery [30]. The concurrent increase in HCT suggests that heat stress (HS) leads to higher blood concentration in sows, potentially increasing blood viscosity and thereby affecting circulation and oxygen transport. Under HS conditions, RBC, HGB, MCV, and MCH levels were significantly decreased in fattening pigs [31], goats [32], broilers [33], and dairy cows [34]. However, in our study, RBC and HGB levels were significantly elevated, consistent with findings from experiments on growing pigs under short-term acute heat stress. This may represent a specific response of sows in early gestation [35]. Additionally, reduced Bas levels indicate suppressed immune system function, leading to decreased sensitivity to potential pathogens and inflammatory responses [36]. Overall, these blood routine indicators suggest that HS during early pregnancy results in decreased blood oxygen content and compromised immune function in sows.

### 4.3. Endocrine Disturbances Adversely Affect Embryonic Development

Heat stress (HS) significantly affects the endocrine system of sows. During early gestation, cortisol levels in sows under HS conditions typically increase, activating the hypothalamic–pituitary–adrenal (HPA) axis to cope with heat stress, which adversely impacts pregnancy [37,38]. In our study, cortisol levels in early gestation sows under HS were significantly elevated, consistent with existing literature [39]. We also observed a significant increase in ACTH expression under HS conditions, likely as a response to stress and to promote cortisol synthesis and secretion [40]. Pregnant sows must bear additional physiological burdens to support fetal development. Additionally, we found decreased IGF-1 levels; insufficient IGF-1 can result in poor embryo development and even death, thereby reducing pregnancy success rates [41]. Therefore, we speculate that high cortisol and low IGF-1 levels under HS may impair embryo implantation and development, leading to developmental delays. In this study, we observed a significant increase in INS levels and a significant decrease in GC levels, indicating that heat stress may disrupt normal glycemic regulation mechanisms. Previous studies have demonstrated that GC plays a crucial role in blood glucose regulation, and its reduced levels can lead to hypoglycemia in sows, thereby affecting the growth and development of embryos during early pregnancy [42,43]. The expression patterns of IGF-1, INS, and GC in early gestation sows were markedly different from those observed under short-term heat stress conditions [44]. T3, as the active form of thyroid hormone, plays a crucial role in regulating metabolic rate and body temperature. TSH, secreted by the pituitary gland, stimulates the thyroid to synthesize and release thyroid hormones. The significant decrease in thyroid hormone levels (T3 and TSH) under heat stress (HS) conditions indicates that HS inhibits thyroid function. This secretion disorder may lead to a reduced metabolic rate in sows, thereby affecting the nutritional supply to the fetus [13,45]. Therefore, we speculate that impaired thyroid function may negatively impact the overall health and reproductive performance of pregnant sows. Additionally, the observed decreases in LH and FSH levels may result from high temperatures directly inhibiting the secretion of reproductive hormones by the hypothalamus and pituitary gland, which seriously affects follicle development and luteogenesis [46,47], particularly detrimental to pregnant sows. Elevated luteinizing hormone (LH) levels under HS conditions may represent a compensatory mechanism to maintain normal luteal function; however, the effectiveness of this mechanism requires further investigation.

### 4.4. Liver Injury Induces Oxidative Stress and Inflammatory Responses

Among blood biochemical indicators, those related to liver function exhibited significant changes under HS conditions. In this study, TP levels in early-pregnancy sows were elevated, which we speculate may be due to the liver’s response to metabolic stress [48]. Elevated GLB levels are associated with immune responses and inflammatory processes, suggesting that the sow’s immune system is combating physiological stress induced by heat stress [49]. The significant increase in TBIL levels beyond the normal range indicates impaired bilirubin metabolism and excretion by the liver [50,51]. Another possible explanation is that high temperatures shorten the lifespan of red blood cells and increase their destruction rate, leading to higher bilirubin production [52]. AST and ALT levels also increased significantly and exceeded the normal range, serving as important markers of hepatocyte injury [53]. Previous studies have demonstrated that elevated AST and ALT levels are associated with reduced production performance in sows, including decreased milk yield and impaired reproductive function [54]. The significant decrease in the A/G ratio further corroborates these findings, indicating reduced albumin synthesis and increased globulin levels, likely due to liver damage-induced decreases in albumin production and enhanced inflammatory responses [55]. This alteration not only reflects a decline in hepatic synthetic function but may also be linked to chronic inflammation and heightened immune activity [56].

Long-term heat stress not only impairs the production performance of sows but also adversely affects their health, particularly by compromising liver antioxidant capacity and enhancing inflammatory responses [57]. HS increases metabolic activity, leading to ROS production and the activation of inflammatory signaling pathways. In this study, we observed that under HS conditions, IL-6 levels in sow livers were significantly elevated, while IL-10 levels were reduced, indicating an increase in pro-inflammatory factors and a suppression of anti-inflammatory responses. These findings further confirm that heat stress triggers hepatic inflammation, consistent with previous research [11,58]. In addition, T-AOC levels were significantly elevated while MDA levels were significantly reduced, indicating that the liver’s antioxidant system was activated under heat stress conditions. This activation successfully mitigated lipid peroxidation and protected cell membrane integrity [59]. SOD, an important antioxidant enzyme, plays a crucial role in reducing oxidative damage risk [60]. In our study, SOD activity was significantly decreased, likely due to the negative impact of heat stress on its synthesis or stability, leading to diminished enzymatic activity. These findings indicate that HS disrupts the redox balance in the liver of early gestation sows, potentially causing oxidative damage.

Further pathological analysis revealed that under HS conditions, the liver tissue of sows exhibited significant pathological changes, including lymphocyte infiltration, punctate necrosis, hepatic sinusoidal dilation, and widened hepatic cord spacing. These changes reflect the activation of the immune system, hepatocellular inflammatory responses, and liver injury [61]. The widened hepatic cord spacing may result from the accumulation of inflammatory cells and altered blood circulation [62,63], leading to irregular tissue arrangement, which can reduce liver functional efficiency and increase the metabolic burden on the liver [11]. These pathological alterations were consistent with elevated levels of TP, GLB, TBIL, AST, and ALT in biochemical indicators, indicating that heat stress significantly impacts liver function. The analysis of apoptotic activity revealed that under HS conditions, the expression level of Bcl-2 in the liver was significantly reduced, leading to an enhanced activity of pro-apoptotic proteins such as Bax. This finding is consistent with previous studies [64,65]. The imbalance between Bcl-2 and Bax indicates increased apoptotic activity, which in turn affects the overall immune response. Additionally, elevated Bax levels typically result in increased mitochondrial membrane permeability, leading to the release of cytochrome c and the activation of downstream apoptosis signaling pathways [66]. A significant increase in CD8-positive cells suggests the activation of cytotoxic T lymphocytes (CTLs), further exacerbating liver cell damage [67]. Heat shock factor (HSF) is activated in response to heat stress (HS), promoting the transcription of HSP genes and leading to a significant upregulation of HSP70 and HSP90 [68]. Proteins such as HSP70 can induce the expression of anti-inflammatory factors, inhibit the phosphorylation of inflammatory transcription factors, and protect cells from damage by suppressing pro-apoptotic signaling pathways, thereby mitigating the inflammatory response [69,70]. In our study, we observed a significant upregulation of HSP70 and HSP90 in liver tissues, indicating that liver cells are actively responding to protein damage and cellular stress induced by heat stress. However, in sows during early pregnancy, despite exposure to long-term chronic heat stress, the expression of HSP70 in the liver increased only twofold. We speculate that this limited upregulation may be attributed to the need for supporting embryo implantation and initial development during early gestation, where the physiological state and metabolic demands differ from those in non-pregnant animals. Consequently, HSP70 expression is not excessively elevated to cope with acute stress conditions.

### 4.5. Multi-Omics Analysis of Liver Oxidative Stress and Inflammation Regulation

Given the observed liver tissue damage and dysfunction, we conducted comprehensive multi-omics analyses of liver tissues, encompassing transcriptomics, proteomics, and metabolomics, to elucidate the molecular mechanisms underlying heat resistance in the liver. The results revealed that differentially expressed genes (DEGs) were primarily involved in oxidoreductase activity and macromolecular biosynthesis processes. KEGG pathway analysis indicated significant enrichment in pathways related to taurine and hypotaurine metabolism, glutathione metabolism, bile secretion, PPAR signaling, and pyrimidine metabolism. Heat stress enhances cells’ ability to cope with oxidative stress by modulating the expression profile of redox enzyme genes and boosting cellular antioxidant capacity [71]. Taurine has been demonstrated to possess anti-inflammatory properties by inhibiting the release of inflammatory mediators such as cytokines and chemokines, thereby mitigating tissue damage and inflammatory responses [72]. Extensive research has shown that taurine also plays a crucial role in antioxidation by increasing intracellular glutathione (GSH) levels, thus protecting cells from oxidative stress-induced damage [73]. Glutathione (GSH) is an important antioxidant that plays a key protective role within cells. Studies have shown that heat stress can increase intracellular oxidative damage and affect the synthesis and degradation of GSH [52,74]. Furthermore, bile acids also play a critical role in alleviating stress-induced damage [75,76]. Heat stress can regulate inflammatory responses through the PPAR signaling pathway; the activation of PPAR can inhibit the expression of pro-inflammatory factors and reduce the severity of inflammatory responses, which is particularly important under heat stress conditions [77]. Therefore, changes in gene expression under HS primarily enhance cellular antioxidant capacity and resistance to inflammatory responses, thereby facilitating heat adaptation.

Heat stress is frequently accompanied by oxidative stress, which can disrupt lipid metabolism in the liver, further impacting cholesterol synthesis and metabolism, and ultimately affecting bile acid synthesis [78]. In this study, we observed that differentially expressed genes (DEGs) and proteins were co-enriched in pathways related to lipid metabolism and bile acid synthesis, indicating that the organism responds to stress through these metabolic adjustments. The GO analysis of the differential proteins revealed significant enrichment in pathways associated with enzyme activity and energy metabolism, including procollagen-proline 4-dioxygenase activity, ATP-dependent DNA helicase activity, sulfotransferase activity, ATP-dependent 3′-5′ DNA helicase activity, and ATPase activity. In this study, we observed that differentially expressed genes (DEGs) and proteins were co-enriched in pathways related to lipid metabolism and bile acid synthesis, suggesting that the organism adapts to stress through these metabolic changes. The GO analysis of the differential proteins revealed significant enrichment in pathways associated with enzyme activity and energy metabolism, including procollagen-proline 4-dioxygenase activity, ATP-dependent DNA helicase activity, sulfotransferase activity, ATP-dependent 3′-5′ DNA helicase activity, and ATPase activity. Differential proteins are primarily enriched in KEGG pathways such as the “Metabolism of xenobiotics by cytochrome P450” and “Basal transcription factors”. The cytochrome P450 (CYP) family is one of the most important drug-metabolizing enzymes in vivo, responsible for the metabolism of various endogenous and exogenous compounds. Under heat stress conditions, increased CYP enzyme activity may lead to the excessive production of reactive oxygen species (ROS), which can trigger liver damage and oxidative stress. Studies have shown that heat stress affects CYP2C and CYP3A, thereby reducing the liver’s ability to metabolize progesterone; this reduction may be a beneficial adaptation for early embryos [79]. Therefore, under heat stress, pregnant sows mitigate the adverse effects of high temperature by regulating the expression of energy metabolism and oxidative stress-related proteins, ensuring the normal growth and development of early embryos.

In this study, differential metabolites were primarily enriched in the biosynthesis pathways of phenylalanine, tyrosine, and tryptophan. It was found that when the temperature reached 27.25 °C, the biosynthesis pathways of phenylalanine and tyrosine were significantly activated, indicating an enhanced synthesis of these amino acids under heat stress [80]. Conversely, under heat stress conditions, the metabolism of tryptophan may be inhibited, thereby affecting its conversion into other bioactive molecules [81]. Functional annotation revealed that the upregulated metabolites included prostaglandin A3, hydroxydodecanoic acid, NAD, and LPCs. NAD is an important coenzyme that plays a critical role in cellular energy metabolism and was found to be upregulated in this study. Studies have shown that NADPH activity is associated with the production of ROS in the liver, which may exacerbate cellular damage caused by heat stress. Additionally, a decrease in NAD levels is linked to increased cellular oxidative stress, potentially leading to liver cell damage and dysfunction [82]. LPCs were significantly upregulated in this study. Studies have shown that LPCs are a major component of oxidized low-density lipoprotein (ox-LDL) and are considered pro-inflammatory factors [83]. In this study, metabolites downregulated in the liver tissues of heat-stressed sows included uracil nucleotide, lysophosphatidylserine (16:0), AMP, and D-(+)-malic acid. Heat stress may lead to the accumulation of uracil by affecting folate metabolism and DNA repair mechanisms, which can result in liver cell damage and dysfunction [84,85]. AMP exhibits a protective effect on the liver during heat stress. By activating AMPK, it can alleviate heat stress-induced liver cell damage and promote cellular repair and regeneration [86]. Empirical studies have shown that supplementation with D-(+)-malic acid effectively mitigates the adverse effects of heat stress on animals. For instance, studies indicate that malic acid supplementation significantly improves liver function indicators and reduces oxidative damage in liver tissue [87]. Additionally, research has found that UMP has a protective effect under heat stress conditions, improving mitochondrial dysfunction caused by heat stress and thereby reducing its negative impact on the liver [88,89]. Combined with transcriptome, proteome, and metabolome multi-omics association analysis, it was found that the liver of early-pregnancy sows under HS primarily regulates the expression of AMP, NAD, UMP, and other metabolites through multiple pathways to activate protective mechanisms. Consequently, the upregulation of NAD and LPCs and the reduction in uracil levels promote oxidative stress and inflammation in the liver. Meanwhile, metabolites with hepatoprotective effects, such as AMP, D-(+)-malic acid, and UMP, are significantly downregulated, further exacerbating liver damage caused by oxidative stress. Therefore, in studies focusing on heat stress, the supplementation of adenosine monophosphate (AMP), D-(+)-malic acid, and uridine monophosphate (UMP) could be explored as a promising strategy to mitigate the adverse effects of heat stress.

## 5. Conclusions

In this study, we successfully established an HS model for early-pregnancy sows and systematically analyzed the effects of HS on these animals from multiple perspectives, including blood physiology, biochemistry, endocrinology, liver histological morphology, and function. Through an integrated analysis of transcriptomics, proteomics, and metabolomics, we revealed the liver’s resistance mechanisms to heat stress. The results demonstrated that under HS, pregnant sows experienced hypoxia, reduced immune function, endocrine imbalance, suppressed thyroid function, inflammation, and oxidative stress. Multi-omics analysis indicated that pregnant sows enhance cellular antioxidant and anti-inflammatory capacities by regulating the expression of genes, proteins, and metabolites related to energy metabolism and oxidative stress, thereby promoting heat adaptation. In this study, we demonstrated that specific metabolites, including AMP, NAD, and UMP, may play a critical role in the mechanisms underlying heat stress resistance. Nevertheless, the causal relationship between these metabolites and heat tolerance remains to be fully elucidated through rigorous experimental validation. Further investigation is warranted to establish conclusive evidence regarding their functional roles in thermotolerance. This study offers valuable insights into strategies for alleviating the adverse impacts of global warming and other extreme climate conditions on early-pregnancy sows. Additionally, it pinpoints specific targets for nutritional intervention, enabling the development of effective strategies to mitigate the detrimental effects of heat stress.

## Figures and Tables

**Table 1:** Routine hematological parameters and blood gas indices of sows in early pregnancy under HS.

Item	Treatments		
	RHC-HY	RHS-HY	*p*-Value
WBC, 10^9^/L	13.55 ± 3.72	14.06 ± 4.35	NS
Neu%	40.23 ± 7.68	45.57 ± 6.54	NS
Lym%	41.17 ± 5.08	41.57 ± 2.59	NS
Mon%	6.33 ± 1.91	6.27 ± 0.35	NS
Eos%	11.63 ± 11.52	6.43 ± 4.48	NS
Bas%	0.63 ± 0.15	0.17 ± 0.12	<0.05
Neu#, 10^9^/L	5.38 ± 1.39	6.25 ± 1.21	NS
Lym#, 10^9^/L	5.46 ± 0.87	5.90 ± 2.05	NS
Mon#, 10^9^/L	0.81 ± 0.07	0.87 ± 0.22	NS
Eos#, 10^9^/L	0.62 ± 0.09	1.46 ± 0.53	NS
Bas#	0.08 ± 0.02	0.02 ± 0.01	<0.05
RBC, 10^12^/L	5.64 ± 0.32	8.18 ± 1.19	<0.01
HGB, g/L	112.33 ± 3.21	137.67 ± 3.515	<0.001
HCT, %	33.70 ± 31.25	44.70 ± 7.03	<0.001
MCV, fL	57.07 ± 5.98	54.67 ± 2.15	NS
MCH, pg	19.30 ± 1.51	18.70 ± 0.80	NS
MCHC, g/L	338.67 ± 9.50	341.67 ± 9.07	NS
RDW-CV, %	17.23 ± 0.06	16.53 ± 0.40	<0.05
RDW-SD, fL	39.83 ± 4.41	36.60 ± 0.78	NS
PLT, 10^9^/L	318.00 ± 6.24	302.00 ± 61.00	NS
MPV, fL	10.47 ± 1.01	10.43 ± 1.12	NS
PDW	19.50 ± 0.75	17.87 ± 1.65	NS
PCT, %	0.19 ± 0.12	0.31 ± 0.04	NS

Note: WBC = white blood cell count; Neu% = (neutrophil percentage); Lym% = lymphocyte percentage; Mon% = monocyte percentage; Eos% = eosinophil percentage; Bas% = basophil percentage; Neu# = neutrophil absolute count; Lym# = lymphocyte absolute count; Mon# = monocyte absolute count; Eos# = eosinophil absolute count; Bas# = basophil absolute count; RBC = red blood cell count; HGB = hemoglobin concentration; HCT = hematocrit; MCV = mean corpuscular volume; MCH = mean corpuscular hemoglobin; MCHC = mean corpuscular hemoglobin concentration; RDW-CV = red cell distribution width—coefficient of variation; RDW-SD = red cell distribution width—standard deviation; PLT = platelet count; MPV = mean platelet volume; PDW = platelet distribution width; and PCT = plateletcrit; Data are expressed as mean ± standard deviation (Std). NS stands for no significant difference.

**Table 2:** Blood gas analysis of sows in early pregnancy under HS.

Item	Treatments		
	RHC-HY	RHS-HY	*p*-Value
Blood pH	7.53 ± 0.17	7.56 ± 0.03	NS
Blood PCO_2_, mmHg	23.60 ± 7.56	30.70 ± 3.64	NS
Blood PO_2_, mmHg	121.67 ± 7.51	50.67 ± 8.39	<0.001
Blood BEecf, mmol/L	2.33 ± 1.15	5.33 ± 1.53	NS
Blood HCO_3_, mmol/L	23.50 ± 1.18	25.83 ± 3.35	NS
Blood TCO_2_, mmol/L	24.00 ± 1.00	28.67 ± 1.53	<0.05
Blood SO_2_%	97.67 ± 3.21	89.67 ± 5.86	NS
Blood Na, mmol/L	137.67 ± 2.52	144.00 ± 1.00	<0.05
Blood K, mmol/L	6.47 ± 0.55	5.23 ± 0.91	NS
Blood Ca, mmol/L	1.18 ± 0.13	1.34 ± 0.03	NS
Blood Hct	31.67 ± 6.51	41.00 ± 1.73	NS
Blood Hb, g/dL	8.83 ± 1.92	13.97 ± 0.58	<0.05

Note: PO_2_ = oxygen pressure, PCO_2_ = pressure of carbon dioxide, BEecf = base excess in the extracellular fluid, HCO_3_ = bicarbonate, TCO_2_ = total carbon dioxide, HCT = hematocrit, and Hb = hemoglobin. Data are expressed as mean ± standard deviation (Std). NS stands for no significant difference.

## Data Availability

All data generated or analyzed during this study are included in this published article and its Supplementary Information Files.

## Supplementary Materials

The following supporting information can be downloaded at https://www.mdpi.com/article/10.3390/antiox14060623/s1: Table S1: Transcriptome data analysis; Table S2: Differential metabolites and gene association analysis; and Table S3: Differential metabolites/gene and protein association analysis. Figure S1: (A) Distribution of peptide segment length ranges; (B) Distribution of the number of unique peptide segments in identified proteins.

## Abbreviations

The following abbreviations are used in this manuscript: heat-stressed early-pregnancy Rongchang sows (RHS-HY), thermoneutral early-pregnancy Rongchang sows (RHC-HY), nicotinamide adenine dinucleotide (NAD), lysophosphatidylglycerol (LPCs), adenosine 5′-monophosphate (AMP), uridine monophosphate (UMP); white blood cell count (WBC); neutrophil percentage (Neu%); lymphocyte percentage (Lym%); monocyte percentage (Mon%); eosinophil percentage (Eos%); basophil percentage (Bas%); neutrophil absolute count (Neu#); lymphocyte absolute count (Lym#); monocyte absolute count (Mon#); eosinophil absolute count (Eos#); basophil absolute count (Bas#); red blood cell count (RBC); white blood cell count (WBC), hemoglobin concentration (HGB); hematocrit (HCT); mean corpuscular volume (MCV); mean corpuscular hemoglobin (MCH); mean corpuscular hemoglobin concentration (MCHC), red cell distribution width—coefficient of variation (RDW-CV); red cell distribution width—standard deviation (RDW-SD), platelet count (PLT), mean platelet volume (MPV), platelet distribution width (PDW), plateletcrit (PCT), oxygen pressure (PO_2_), pressure of carbon dioxide (PCO_2_), base excess in the extracellular fluid (BEecf), bicarbonate (HCO3), total carbon dioxide (TCO2), hematocrit (HCT), hemoglobin (Hb), albumin (ALB), total protein (TP), globulin (GLB), albumin-to-globulin ratio (A/G), total bilirubin (TBIL), gamma-glutamyl transferase (GGT), aspartate aminotransferase (AST), alanine aminotransferase (ALT), alkaline phosphatase (ALP), total bile acid (TBA), triiodothyronine (T3), thyrotropin (TSH), free thyroxine (FT4), adrenocorticotropic hormone (ACTH), luteinizing hormone (LH), follicle-stimulating hormone (FSH), glucagon (GC), angiotensin I (Ang-I), angiotensin II (Ang-II), estradiol (E2), prolactin (PRL), immunoglobulin G (IgG), aldosterone (ALD), insulin (INS) and insulin-like growth factor (IGF-1), interleukin-6 (IL-6), tumor necrosis factor-α (TNF-α), interleukin-1β (IL-1β), interleukin-10 (IL-10), total antioxidant capacity (T-AOC), malondialdehyde (MDA), and superoxide dismutase (SOD).
