# Supplementary figures and images for: Multi-Omics Analysis of Chronic Heat Stress-Induced Biological Effects, Liver Injury, and Heat Tolerance Mechanisms via Oxidative and Anti-Inflammatory Pathways in Early-Pregnancy Sows
Source: Antioxidants (Basel). 2025 May 23;14(6):623. doi: 10.3390/antiox14060623 (PMC12189682; doi:10.3390/antiox14060623)

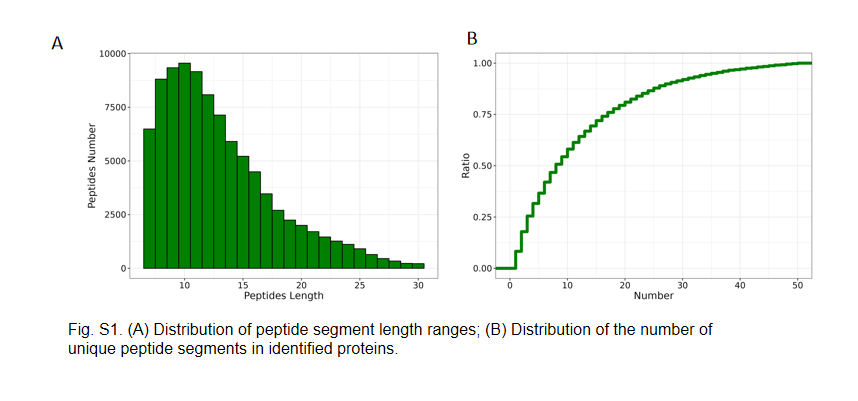

Supplement: Supplementary file 1 [file antioxidants-14-00623-s001.zip › Figure S1.png]
